# A critically co-endangered feather louse *Forficuloecus pezopori* n. sp. (Phthiraptera: Philopteridae) detected through conservation intervention for the western ground parrot *Pezoporus flaviventris* (Psittaculidae)

**DOI:** 10.1016/j.ijppaw.2024.100931

**Published:** 2024-04-01

**Authors:** Storm Blas Martin, Sarah Keatley, Alisa Wallace, Rebecca J. Vaughan-Higgins, Amanda Ash

**Affiliations:** a Centre for Sustainable Aquatic Ecosystems, Harry Butler Institute, Murdoch University, Perth, Western Australia, Australia; b Centre for Biosecurity and One Health, Harry Butler Institute, Murdoch University, Perth, Western Australia, Australia; c Wildlife Hospital, Taronga Western Plains Zoo, Dubbo, New South Wales, Australia; d Centre for Terrestrial Ecosystem Science and Sustainability, Harry Butler Institute, Murdoch University, Murdoch University, Perth, Western Australia, Australia; e Veterinary Department, Perth Zoo, Perth, Western Australia, Australia

**Keywords:** Co-extinction, Psocodea, Ischnocera, Lice, Endemic, Translocation, Parasite conservation

## Abstract

*Forficuloecus pezopori* Martin, Keatley & Ash n. sp. from the western ground parrot *Pezoporus flaviventris* North, 1911 (Psittaculidae) is proposed based on combined evidence from morphology and *COI* mitochondrial DNA. Phylogenetically, the new species is closest to its two known congeners from Western Australia: *F*. *josephi* Price, Johnson & Palma, 2008 from Bourke's parrot *Neopsephotus bourkii* (Gould, 1841) and the scarlet-chested parrot *Neophema splendida* (Gould, 1841), and *F*. *palmai* Guimarães, 1985 from the Australian ringneck parrot *Barnardius zonarius* (Shaw, 1805). Morphologically it is distinguishable by abdominal chaetotaxy and characters of the male genitalia, and is most similar to *F*. *josephi* and *F*. *greeni* Guimarães, 1985; the latter has no representative sequence data. *Forficuloecus pezopori* is the eleventh species of its genus and the only metazoan parasite known from *P*. *flaviventris*, which is among Australia's most endangered vertebrates. The new louse is apparently restricted to *P*. *flaviventris* and is therefore co-endangered, facing at least the same likelihood of extinction as its host. We recommend ongoing translocation and field monitoring efforts for *P*. *flaviventris* include monitoring but not treatment for lice infestations in otherwise healthy individuals, and that the care management plan for captive *P*. *flaviventris* considers that *F*. *pezopori* is similarly imperilled.

## Highlights

• The first metazoan parasite known from the western ground parrot.
• The new louse is likely restricted to its host and thus also critically endangered.
• Conservation interventions for the parrot ought to consider the louse.
• Host interventions are opportunities to discover and conserve parasites.

## Graphical abstract

**Figure:**
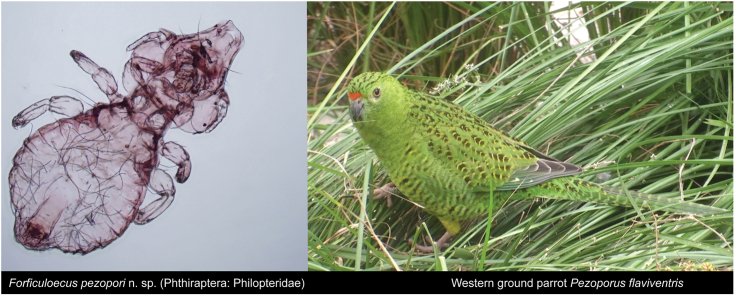

## 1 Introduction

Parasites are scarcely considered in conservation management (Carlson et al., 2020a, Carlson et al., 2020b; Lymbery and Smit, 2023). Nevertheless, essentially all conservation efforts likely impact parasites, regardless of whether those taxa are known or the outcomes valued. Stabilising or reversing a negative population trend for an endangered host species will often elicit a comparable outcome for its dependent parasite taxa (Lymbery and Smit, 2023). However, some interventions will be too late to benefit dependent parasite taxa, because host-specific symbionts are always more endangered than their host and may disappear sooner as density dependence pressures increase with host decline (Lymbery and Smit, 2023; Mackenzie and Pert, 2018; Wood et al., 2023). Interventions involving veterinary checks, captive breeding programs or translocations may even expedite extinction for dependent parasites, either through intentional removal or disruption of life cycles and transmission (Dunn et al., 2009; Lymbery and Smit, 2023; Milotic et al., 2020; Rózsa and Vas, 2015).

Among Metazoa, parasites likely constitute most of the richness yet to be characterised, most of the taxa threatened with extinction, and most of the taxa which have been or will be lost in the Anthropocene without becoming known to science (Carlson et al., 2020a, Carlson et al., 2020b; Dunn et al., 2009; Poulin and Morand, 2000). Simultaneously, vertebrates receive the majority of species-oriented conservation effort and are disproportionately significant as parasite hosts. Yet many vertebrate species remain entirely unsurveyed for parasite diversity, and many more only scantily so, or only for certain taxonomic groups or from few discrete localities (Cribb et al., 2016). Endangered vertebrates therefore represent imminent loss of parasite fauna. In this context, conservation interventions frequently provide the best opportunity for detection and collection of novel parasite taxa associated with endangered host species. Indeed, several parasite species have been detected and characterised through host conservation efforts, including both from captive animals and through monitoring and handling of animals in the field (e.g. Ash et al., 2017; Chiangkul et al., 2021; 2017; Ingelbrecht et al., 2022; Northover et al., 2019; Sychra, 2006).

The western ground parrot or kyloring *Pezoporus flaviventris* North, 1911 (Psittaculidae) is one such imperilled vertebrate for which metazoan parasites are entirely unknown. It is endemic to the South-West Australian Floristic Region and, following progressive decline and range contraction across several decades, is now restricted to a single population estimated at less than 150 individuals with an estimated extent of occurrence of 580 km^2^ (Burbidge et al., 2016; Comer et al., 2021). Although not yet listed by the IUCN Red List, it is deemed critically endangered under both Australian and Western Australian legislation (Comer et al., 2021, Department of Parks and Wildlife, 2014), and is among the nine most threatened Australian vertebrate taxa, likely extirpated from > 99% of its past potential habitat (Ward et al., 2022) and with estimated greater than even odds of extinction by 2041 (Garnett et al., 2022).

Conservation intervention effort and investment for the western ground parrot is considerable (Comer et al., 2021, Department of Parks and Wildlife, 2014). Efforts to establish a captive breeding program have been ongoing since 2009 (Burbidge et al., 2016, 2018) and several individuals were recently translocated from the sole remaining wild population to a locality from which the species was previously recorded (Stokes et al., 2021).

Here we report on the first metazoan parasite known from *P*. *flaviventris*, a biting louse discovered through these direct conservation interventions for its host.

## 2 Materials and methods

### 2.1 Procurement of specimens and host identity

An infestation of lice was detected on two captive western ground parrots housed at Perth Zoo in 2020. These two individuals were translocated to Perth Zoo from a captive facility in southern Western Australia in 2014, originally captured from Cape Arid National Park in 2009 and 2010. Other western ground parrots were translocated from Cape Arid National Park to Perth Zoo in 2018; these might have been the source of infestation. Additional louse specimens were collected from three western ground parrots during a wild-to-wild translocation from Cape Arid National Park to an area east of Albany (Stokes et al., 2021), confirming that the western ground parrot is indeed the host *versus* the possibility of contamination from other captive birds.

Here, we consider *P*. *flaviventris* a distinct species *versus* a subspecies of the eastern ground parrot *P*. *wallicus* (Kerr, 1792), following recent evidence (Joseph et al., 2011; Murphy et al., 2011) and recognition in the Australian Faunal Directory and primary literature (e.g. Hamilton et al., 2017, Molloy et al., 2020, Newbey, 2016, Thomas et al., 2020, White et al., 2023; see also Burbidge et al., 2016). The western ground parrot is not currently recognised at the species level by prominent bird checklists (BirdLife International, 2015; Clements et al., 2023).

### 2.2 Morphological study

Specimens for morphological study were macerated and decoloured in 20% aqueous potassium hydroxide, neutralised in 10% aqueous acetic acid, stained with acid fuchsin, dehydrated through a graded ethanol series, cleared in clove oil and whole mounted without dissection in Canada balsam diluted with methyl salicylate, modified from Palma (1978).

Morphometric data were taken via live feed with cellSens Standard v1.13, from an Olympus BX50 microscope with Nomarski interference contrast and an Olympus DP71 digital microscope camera and UCMAD3 adaptor (Olympus Inc., Tokyo, Japan). Line drawings were traced *via* camera lucida, from an Olympus BHA phase contrast microscope, and then digitised in Adobe Illustrator CC using a Kamvas 20 drawing tablet (Huion, China). Descriptions and terminology are especially influenced by reference to Clay (1951), Najer et al. (2016), Symmons and Hindle (1952), and Valim and Palma (2013). Type-material has been archived in the entomology collection of the Western Australian Museum, Perth (WAM), and genetic data in GenBank (GB). In compliance with article 8.5 of the amended 2012 version of the International Code of Zoological Nomenclature (ICZN, 2012), details of new taxa have been submitted to ZooBank.

### 2.3 Molecular barcoding and phylogenetic study

#### 2.3.1 Extraction, amplification and sequencing

Novel sequence data were generated for *COI* mtDNA. Genomic DNA was extracted from five whole specimens (4 female, 1 male, including from captive and wild birds) using a QIAGEN (Hilden, Germany) DNeasy blood and tissue extraction kit. The target marker was amplified by conventional PCR, using the primers L6625 (5′- CCGGATCCTTYTGRTTYTTYGGNCAYCC-3′) and H7005 (5’ -CCGGATCCACNACRTARTANGTRTCRTG-3′) of Hafner et al. (1994) and the following cycle schedule of Johnson and Clayton (2000) as per Johnson et al. (2001): an initial denaturation of 2 min at 94 °C, 35 cycles of 30 s each at 94 °C, 46 °C and 72 °C, and a final extension of 7 min at 72 °C. The PCR used 50 μl reaction volumes comprising 4 μl unquantified genomic DNA, 3 μl per primer at 10 μM, 2 μl dNTPs, 6 μl cresol red visualisation dye, 24.6 μl purified injection water, 3.9 μl MgCl_2_ at 25 mM and 1 μl T. *aquaticus* DNA polymerase at 5.5 units/μl with 2.5 μl 10 × reaction buffer (Fisher Biotec Australia).

Amplicons were visualised via electrophoresis using 1.5% agarose gels stained with SYBR Safe (Invitrogen, California, USA) and purified using Agencourt AMPure magnetic bead purification system (Beckman Coulter, California, USA). Sanger sequencing was completed by the Western Australian State Agricultural Biotechnology Centre (SABC) at Murdoch University, using an ABI Prism™ BigDye v3.1 Cycle Sequencing Kit (Applied Biosystems, California, USA) and an ABI 3730 96 capillary machine. Forward and reverse DNA strands were sequenced using the amplification primers. Individual reads were examined and trimmed, and contiguous sequences were assembled, in Geneious v10.2.2.

#### 2.3.2 Phylogenetic analyses

Novel *COI* mtDNA were aligned against all comparable data representative of species of *Forficuloecus* available at GenBank, using the implementation of MUSCLE (Edgar, 2004) in MEGA 11 (Tamura et al., 2021), with default parameters, including UPGMA clustering for both iterations. No ambiguously aligned regions were detected. Interspecific genetic differences were examined with a pairwise distance matrix. Phylogenetic relationships were reconstructed via maximum likelihood analysis using the implementation of RaxML v8.2.12 (Stamatakis, 2014) in the CIPRES portal (Miller et al., 2010), and included comparable data for *Theresiella gemina* Guimarães, 1971 as the outgroup. The analysis assumed the GTR + Γ model of nucleotide substitution and otherwise used default parameters; the best model selected by jModelTest v2.1.10 (Darriba et al., 2012) was TIM3 + Γ + I under the Akaike Information Criterion (AIC) and TrN + Γ under the Bayesian Information Criterion (BIC). The maximum likelihood analysis ran 400 bootstrap pseudoreplicates as determined with the majority rule bootstopping criterion (autoMRE) (Pattengale et al., 2009).

## 3 Results

### 3.1 Pathology in captive animals

Lice were detected on two captive *P*. *flaviventris* individuals associated with skin irritation and feather abnormalities (fraying) likely from the chewing action of the lice or pruritus from the host. The lice were found on the back of the head and neck and were easily removed. The skin on the head area was scurfy but the rest of the feathering appeared normal. Based on these clinical signs and because the infested individuals were co-habiting with other individuals for breeding, treatment was initiated with Ivermectin (Avimecâ Vetafarm, Wagga Wagga, Australia).

### 3.2 Molecular and phylogenetic results

**Fig. 1:**
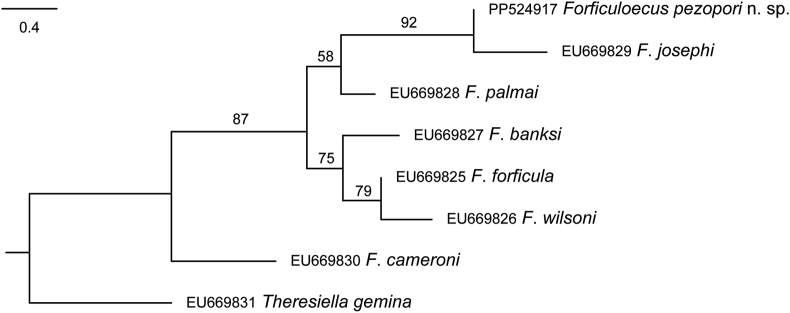
Inferred phylogeny for species of *Forficuloecus* based on maximum likelihood analysis of *COI* mtDNA. Nodal support are from 400 bootstrap replicates. The scale-bar indicates the expected number of substitutions per site.

Novel *COI* mtDNA generated from five lice specimens were identical, and distinct relative to comparable data for species of *Forficuloecus* available at GenBank. In the maximum likelihood phylogenetic analysis the species represented by the novel data resolved together with species of the *F*. *forficula* (Piaget, 1871) group, and was sister to *F*. *josephi* Price, Johnson & Palma, 2008 and then *F*. *palmai* Guimarães, 1985 (Fig. 1). The novel data differ from comparable data for *F*. *josephi* at 49 of 379 (13%) base-positions; the smallest interspecific distinction in *COI* among the taxa considered is 44 base-positions between *F*. *forficula* and *F*. *wilsoni* Price, Johnson & Palma, 2008.

### 3.3 Taxonomy

**Fig. 2:**
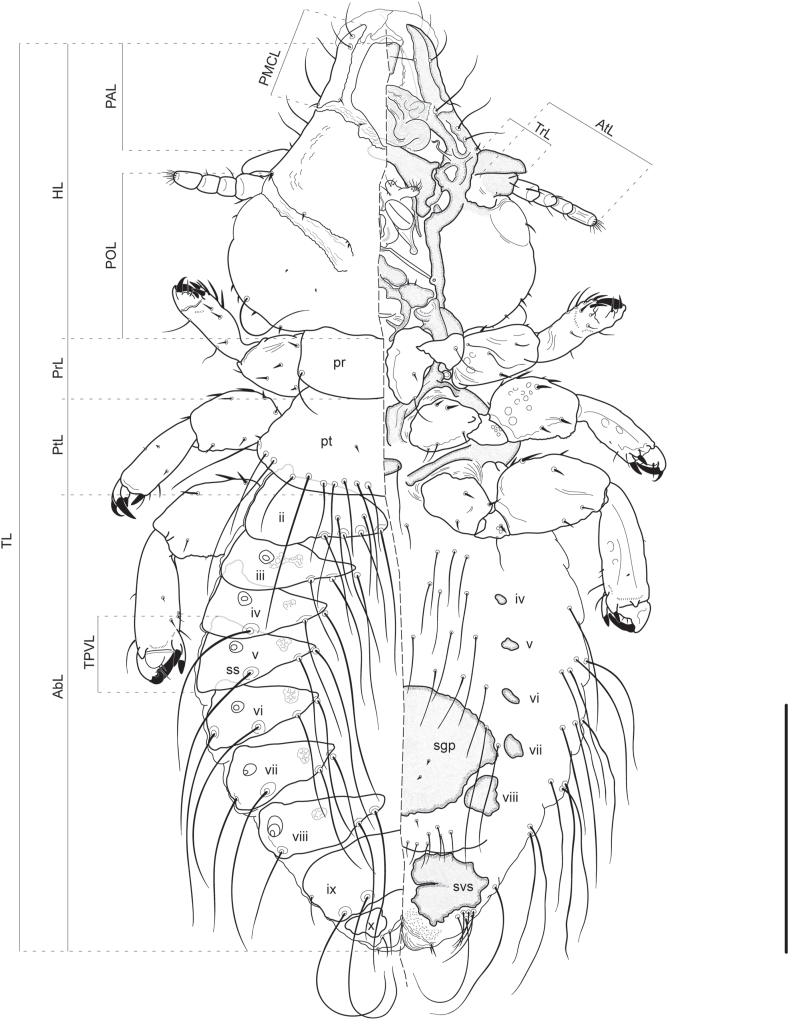
*Forficuloecus pezopori* Martin, Keatley & Ash n. sp. habitus, female allotype, dorsal (left) and ventral perspective. Some variably absent setae added from reference to paratypes. Tergites and sternites labelled with Roman numerals. Abreviations: ms, mesosternum; pn, pronotum; ps, prosternum; pt, pterothorax; ss, spiracular seta; sgp, subgenital plate. Scale bar: 250 μm.

**Fig. 3:**
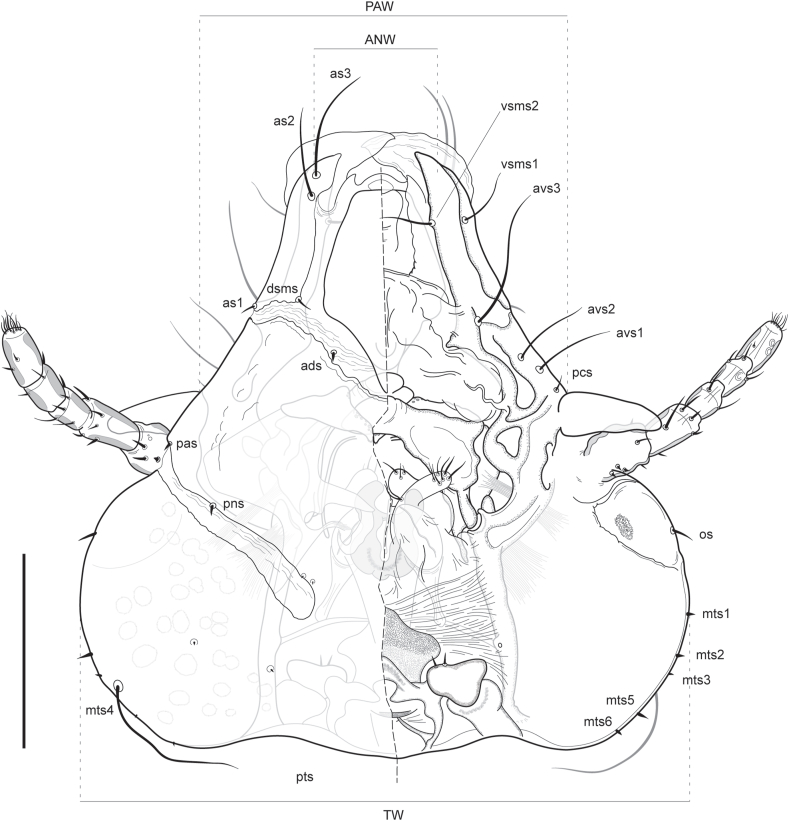
*Forficuloecus pezopori* Martin, Keatley & Ash n. sp. head, female paratype, dorsal (left) and ventral perspective. Abbreviations: *ads*, anterodorsal seta; *as*, anterior seta; *avs*, anteroventral seta; *dsms*, dorsal submarginal seta; *mts*, marginal temple seta; os, ocular seta; *pas*, preantennal seta; *pcs*, preconal seta; *pns*, postnodal seta; *pts*, posterior temple seta; *vsms*, ventral submarginal seta. Scale bar: 200 μm.

**Fig. 4:**
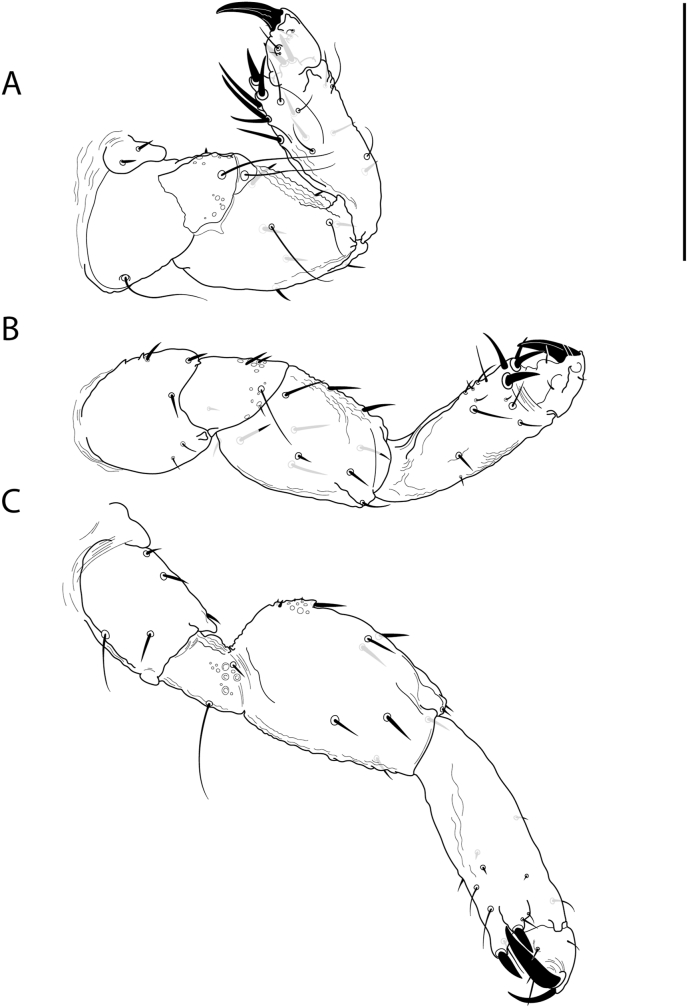
*Forficuloecus pezopori* Martin, Keatley & Ash n. sp. male holotype, ventral perspective, A. proleg, B. metaleg, C. mesoleg. Scale bar: 200 μm.

**Fig. 5:**
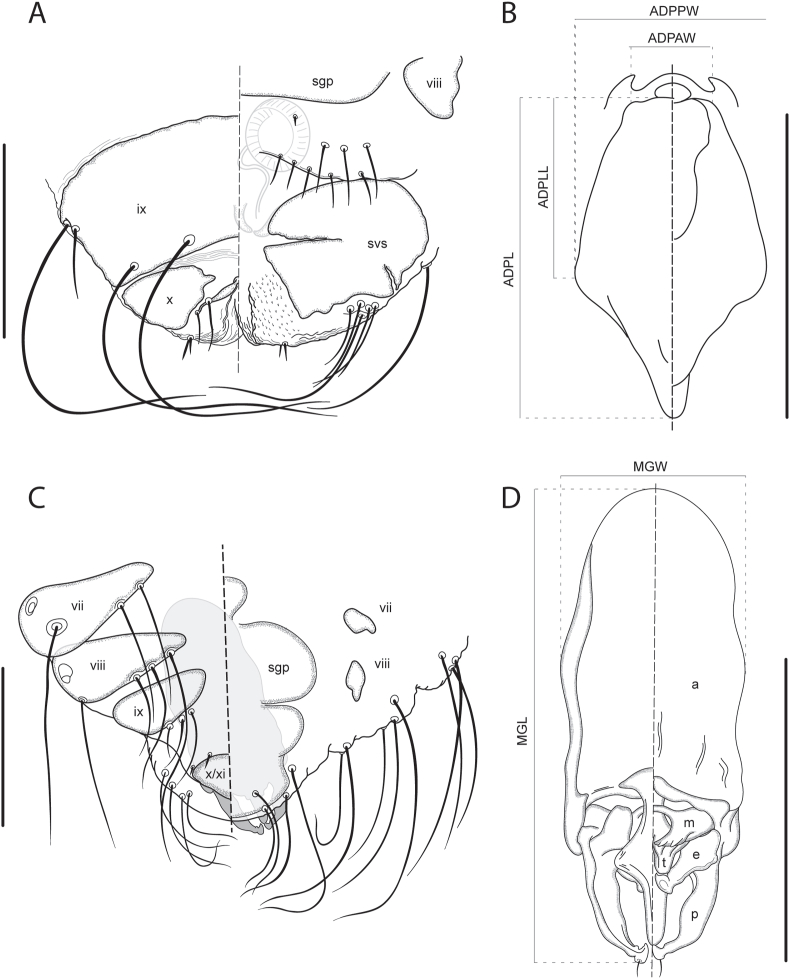
*Forficuloecus pezopori* Martin, Keatley & Ash n. sp., dorsal (left) and ventral perspective, A. female allotype posterior terminus with egg, B. female alloptype anterior dorsal plate, C. male holotype posterior terminus, D. male holotype terminal genitalia. Tergopleurites and sternopleurites labelled with Roman numerals. Abbreviations: a, basal apodeme; e, endomere; m, mesosomal plate; p, paramere; sgp, subgenital plate; svs, subvulvular sclerite; t, telomere. Scale bars: 200 μm.

*Forficuloecus pezopori* Martin, Keatley & Ash n. sp. (Fig. 2, Fig. 3, Fig. 4, Fig. 5)

*Classification*: Phylum Arthropoda: Class Insecta: Order: Psocodea: Parvorder: Phthiraptera: Family Philopteridae: Genus *Forficuloecus* Conci, 1941.

*Synonyms*: none.

*Type-host*: *Pezoporus flaviventris* North, 1911 (Psittaculidae), western ground parrot.

*Type-locality*: Cape Arid National Park, Western Australia.

*Other localities*: Perth Zoo (hosts originally from Cape Arid National Park).

*Site of infection*: Feathers on head and neck.

*Prevalence and intensity*: Detected on 2 of 8 (25%) captive birds and 3 of 7 wild birds (43%) at low intensity. It is possible a single bird might have been the original source of the infestation on the captive birds.

*Material examined/deposited*: Holotype (WAM E117337), allotype (WAM E117338), and nine paratypes (WAM E117339–47).

*Representative genetic sequences*: Five identical replicates of *COI* mtDNA (GB PP524917).

*ZooBank registration*: LSID: https://zoobank.org/urn:lsid:zoobank.org:act:3A5ABCA8-537E-4738-B627-2B4152FE8B81.

*Etymology*: This species is named for its host *Pezoporus flaviventris*.

*Description*. Based on 2 male and 9 female specimens. Measurements in Table 1, abdominal chaetotaxy in Table 2. Body somewhat dorsoventrally flattened. Head non-circumfasciate, broadly triangular, roughly as long as wide; preantennal region with concave lateral margins, terminates in forceps-like projections extending beyond dorsal anterior plate and surrounded by substantial hyaline. Preantennal marginal carina interrupted medially and laterally; lateral interruption complete, anterior of mandibles at level of broadest part of dorsal anterior plate and midlevel of pulvinus. Dorsal anterior plate with narrow and almost straight anterior margin, becomes increasing broad posteriorly to level of lateral suture, then narrows with slightly concave lateral margins to rounded, posterior, double point which partially overlaps mandibles; ventral anterior plate narrower than and less than half the length of dorsal anterior plate. Anterior hyaline margin rounded, convex, prominent, with anteriorly recurved lateral points. Postnodal suture broad, incomplete, run posteromedially from antennae base. Pulvinus extends anteriorly to midlevel of dorsal anterior plate.

**Table 1:** Morphometric data from the type-series of *Forficuloecus pezopori* Martin, Keatley & Ash n. sp. as range with mean and standard deviation in parentheses. Measurements (and most abbreviations) are consistent with those defined by Valim and Palma (2013) and Najer et al. (2016), and depicted in Fig. 2, Fig. 3, Fig. 5. Morphometric data are in micrometres (μm) expressed as range followed by mean and standard deviation in parentheses.

Morphometric	Male (n = 2)	Female (n = 9)
ADPL anterior dorsal plate length	174–175 (175, 1)	153–228 (200, 22)
ADPLL anterior dorsal plate lateral length	97–108 (103, 8)	82–126 (114, 14)
ADPAW anterior dorsal plate anterior width	46–49 (48, 2)	21–63 (50, 13)
ADPPW anterior dorsal plate posterior width	108–112 (110, 3)	110–141 (127, 11)
AbL abdomen length	590–663 (627, 52)	677–1011 (904, 111)
AbW abdomen width	636–653 (645, 12)	665–972 (828, 92)
ATL antenna length	195–206 (201, 8)	196–279 (241, 25)
AF1L antenna flagellomere I length	35–37 (36, 1)	32–47 (40, 6) (n = 8)
AF2L antenna flagellomere II length	33–35 (34, 1)	26–46 (35, 6) (n = 8)
AF3L antenna flagellomere III length	52–54 (53, 1)	46–63 (55, 6) (n = 8)
APL antenna pedicel length	42–47 (45, 4)	43–66 (58, 7) (n = 8)
ASL antenna scape length	39–48 (44, 6)	43–64 (55, 7) (n = 8)
HL head length	486–505 (496, 13)	466–625 (572, 51)
MGL male genitalia length	314–319 (317, 4)	–
MGW male genitalia width	115–123 (119, 6)	–
NW anterior notch width	90–93 (92, 2)	100–157 (126, 19)
PAL preantennal length	180–181 (181, 1)	134–236 (202, 35)
PAW preantennal width	307–319 (313, 8)	308–413 (368, 32)
POL postanternnal length	268–278 (273, 7)	258–341 (311, 31)
PMCL pre marginal carina length	143–144 (144, 1)	114–184 (159, 24)
PrL prostonum length	101–105 (103, 3)	83–137 (124, 17)
PrW prostonum width	256–293 (275, 26)	268–393 (344, 38)
PtL pterothorax length	127–139 (133, 8)	164–268 (201, 32)
PtW pterothorax width	400–439 (420, 28)	406–578 (507, 54)
SGPW subgenital plate width	–	372–425 (389, 25) (n = 4)
TL total length	1320–1385 (1353, 46)	1386–2045 (1796, 190)
TPVL tergal plate V length	94–105 (100, 8)	149–174 (159, 11) (n = 5)
TrL trabecula length	82–93 (88, 8)	96–134 (112, 12)
TrW trabecula width	33–38 (36, 4)	41–57 (51, 5)
TW temple width	489–512 (501, 16)	478–669 (592, 63)
ED egg diametre	–	75–97 (82, 9) (n = 5)

**Table 2:** Abdominal chaetotaxy of *Forficuloecus pezopori* Martin, Keatley & Ash n. sp. compared with its closest morphological congeners, interpreted from descriptions and illustrations in Guimarães (1985) and Price et al. (2008). Counts indicate the number of setae either side of the midline. Setae are divided into: tergocentral (tc), dorsal and medial of the spiracles; tergolateral (tl), dorsal and lateral of the spiracles; sternocentral (sc), ventral and medial of the sternopleurites; sternolateral (sl), ventral and lateral of the sternopleurites. Where the chaetotaxy differs between males and females a comma separates the sexes, with the male count first. Price et al. (2008) did not describe or figure sternolateral setae. The spiracular setae of *F*. *pezopori* are included here in the tergolateral count (and not in the tergocentral count) for consistency with the congeneric taxa, despite being medial of the spiracles (see Remarks). The plus symbol (+) indicates an additional row of setae.

	*F*. *pezopori* n. sp.				*F*. *josephi*			*F*. *greeni*			
	tc	tl	sc	sl	tc	tl	sc	tc	pt	sc	ps
II	4–5 + 1, 3–5 + 0–2	0	1	0	2–3	0	1	3–4	0	1	0
III	2–3, 3–4	0	3, 2–3	0	2, 2–3	0	1–2	2–3	0	2, 2–3	0
IV	2	1	3–4, 3	1	2, 2–3	0	2	3–4	1	2–3	1
V	2	1	2–3, 3	3, 4	2, 2–3	2	2	3–4	1	2–3, 3	2
VI	2	2	2–3	3	2	2	2–3	3–4	2	0–1, 3	1, 2
VII	2	2	0, 2	2	2	2	2–3	3–4	2	0–1, 2–3	1
VIII	2–3	1	0, 8–10	2	1–2, 2	2	0, 5	2–3	2	0,7–10	1
IX	2–3, 2	0, 1	0	1	3, 2	2	0	3, 1	2	0	1

Trabecula short, broad, taper to rounded point. Antennae monomorphic, short; socket shallow, to base of trabeculum; scape short, broad, almost as wide as long, with three short, distal, marginal, spine-like setae, two dorsal and one ventral, and one dorsal and two to three ventral, minute, posteroproximal, spines; pedicel rectangular, similar in length to scape, not fused to flagellomeres, with eight setae; flagellomeres I shorter than pedicel, with six setae, flagellomere II similar in length to flagellomere I, with four setae; flagellomere III longer, with three setae and distal sensilla trichoidea.

Head chaetotaxy. First anterior seta *as1* short, dorsomarginal, immediately anterior to lateral interruption of marginal carina by dorsal preantennal suture. Second anterior setae *as2* long, submarginal on marginal carina at level of anterior margin of dorso-anterior plate. Third anterior setae *as3* long, anterior to *as2*, at level of anterior hyaline margin. Dorsal submarginal seta *dsms* short, medial of *as1*, at posterior dorsomedial margin of marginal carina where lateral and medial preantennal sutures meet. Anterior dorsal setae *ads* short, submedial on lateral suture, postero-medial relative to *dsms*. Preantennal setae short *pas*, dorsal, at anterior base of antennae scape. Preconal setae *pcs* short, ventral, anterior to conus base. First anterior ventral setae *avs1* long, submarginal, anterior to *pcs*. Second anterior ventral setae *avs2* long, submarginal, anterior to *avs1*. Third anterior ventral setae *avs3* longer than and anteromedial of *avs1* and *avs2*, at level of dorsal preantennal lateral suture. First ventral submarginal setae *vsms1* long, immediately posterior relative to *as2*. Second ventral submarginal setae *vsms2* medial of *vsms1* long, anterior of pulvinus on inner margin of forceps-like preantennal structure. Mandibular setae apparently absent, no setae on surface of pulvinus. Postnodal setae *pns* short, dorsal, strongly submedial, at level of eye, positioned on anterior margin of postnodal suture. Occipital setae short, lateral, on lens of eye. Marginal temple setae pairs six; *mts1–3* and *mts5-6* short, marginal; *mts4* long, dorso-submarginal. Additional setae up to five each side, all minute, often with two or three on temple surface and two near to anteromedian margin of postnodal suture.

Thorax shorter and narrower than head, wider than long. Pronotum roughly rectangular, with single pair of long, posterolateral setae. Prosternum with two pairs of short, spine-like, submedial, anterior setae posterolateral of gula. Mesosternum without setae, with pair of posterolateral spiracles. Pterothorax wider than long, trapezoidal with convex lateral and posterior margins, with 9–11 pairs of long, dorsal, posteriorly submarginal setae and one pair of short posterolateral spine-like setae, sometimes with one pair of small submedial setae at midlevel. Pterothoracic sternal plate small, with single pair of sublateral setae.

Leg coxae short, broad; trochanters shorter than coxae; femurs broad, longer than coxae; tibiae narrower than femurs, similar to femurs in length in proleg and longer than femurs in metaleg and mesoleg; tarsae short. Metaleg longer than proleg. Mesoleg longest. Leg chaetotaxy of holotype: Proleg coxae with two anteroventral spines and one long posteroventral seta; trochanters with two long, ventral, distal marginal setae and one or more minute anterior spines and bosses; femurs with two long ventral setae and seven dorsal spine-like setae; tibiae with four large ventromarginal spines, one proximal and paired distal, moderately long anterior setae, three ventrolateral moderately long seate, four moderately long dorsal spines; tibiae with large distal claw, two pairs of smaller ventrolateral claws, and few short setae. Metaleg coxae with one anteroventral spine, two ventral and distal marginal spines, and two short posteroventral setae; trochanters with two anteroventral spines and two ventral and distal marginal setae, one short and the other long; femurs with ten spines; tibiae with paired large ventrolateral spines, four short to moderate ventrolateral setae, seven ventral setae; tarsae with large distal claw, second smaller distal claw, paired ventral claws, few setae. Mesoleg coxae with three anteroventral setae, one moderate length ventral seta, and one long posteroventral seta; trochanters with one long posteroventral seta and one short, ventral, distal marginal seta; femurs with few proximal, anterior, minute spines and bosses, two anterior spines and two short distal marginal and anterior spines, three ventral and two dorsal spines and one dorsal and distal marginal spine; tibiae with eight short to moderate setae; tarsae with large distal claw, second smaller distal claw, paired ventral claws, few setae;

Abdomen with nine apparent segments numbered II–X/XI, widest at segment V, with pair of sublateral dorsal spiracles each on III–VIII. Tergopleurites II–VIII divided in both sexes; tergocentral setae in single posteromedial row on either side (II 3–5, III 2–4, IV–VII 2, VIII 2–3), often with a second, anterior row of 1–2 on II; spiracular setae one each either side of IV–VIII, posteromedial of spiracle, stronger than and lateral to tergocentral setae; tergolateral setae one each either side on VI–VII. Sternopleurites absent from anterior sterna, greatly reduced and submedial in IV–VIII; sternocentral setae submedial, medial of sternopleurites, shorter and weaker than tergocentral setae, in single row on either side (II 1, III 2–3, IV 3–4, V–VI 2–3); sternolateral setae stronger and longer than sternocentral setae, in ventrolateral cluster (IV 1, V 3–4, VI–VIII 2, IX 1), absent on II–III.

Male. Abdomen shorter and rounder relative to female; posterior extremity protuberant and bluntly rounded. VII–VIII without sternocentral setae. IX–XI difficult to interpret in available specimens; IX seemingly with tergopleurites divided with 2–3 tergocentral setae and one sternolateral seta either side; X/XI seemingly with tergopleurites fused with two, minute setae along anterior margin, four dorsal and posteroterminal setae, and four ventral and posteroterminal setae, either side. Ventral subgenital plate difficult to trace, seemingly spans segments VII–X. Male genitalia elongate; basal apodome extends anteriorly to segment VII, rounded proximally, sclerotised dorsolaterally along most of length, distal margin strongly sclerotised ventrally and weakly so dorsally; parameres long, with almost straight external margin to level of end of penis then sharply curved inward, with prominent hooked or recurved distal tip and single subterminal seta; endomeres ventral to and about half length of parameres, stout, directed posteromedially, with hooked distal tip, apparently without setae or tubercles; telomeres short, blunt-ended, immediately submedial, directed posteriorly, ventral to but largely not overlapping endomeres; mesosomal plate ventral to telomeres, with nearly circular medial aperture arising from incomplete posterior margin, anterior sclerotised margin narrow and separated from posterior sclerotised margin of basal apodeme, posterior margin strongly sclerotised with two to three submarginal tubercles; penis elongate, tapers gently distally.

Female. Abdomen ovate; posterior extremity bluntly rounded. IX with tergopleurites entire, prominent with two, long medioposterior setae and two anterolateral setae, one short and one long, either side; sternopleurites fused with those of X to form subvulvular sclerites, with one sternolateral seta either side. X/XI with tergopleurites separate, reduced, slightly submedial, with two short, dorsal, submedial setae flanking genital opening, a cluster of 5–6 longer, posterolateral setae, and two short, spine-like, terminal setae, one dorsal and one ventral, either side. Ventral subgenital plate clamshell shape, spans roughly half abdomen width on VI–VIII, with two pairs of minute, submedial spines. Subgenital fold on VIII between subgenital plate and subvulvular sclerites, with 9–11 short setae either side with one row of 4–5 along margin of fold, a cluster of 3–4 anterolateral to that row, and a single, smaller seta submedial and just posterior to the subgenital plate.

#### 3.3.1 Remarks

Price et al. (2008) divided species of *Forficuloecus* into two species groups, the *F*. *forficula* species group and the *F*. *meinertzhageni* Guimarães, 1974 group. *Forficuloecus pezopori* is consistent with the *F*. *forficula* group, defined for species in which both sexes have one (*vs* two) long seta on each lateral temple margin, the male has a conspicuously elongate and tapered penis, and the subgenital plate of the female has few setae grouped around inwardly pointed sclerites. This group includes: *F*. *forficula*, *F*. *banksi* Price, Johnson & Palma, 2008, *F*. *greeni* Guimarães, 1985, *F*. *josephi*, *F*. *palmai* and *F*. *wilsoni*. *Forficuloecus pezopori* is among the smallest species in this group together with *F*. *greeni* and *F*. *josephi*. It is most similar to those species, and keys to *F*. *josephi* using Price et al. (2008).

The abdominal chaetotaxy is useful for distinguishing *F*. *pezopori*. Price et al. (2008) distinguished *F*. *josephi* from all other species of the group by fewer tergocentral setae, with at most two setae medial of the spiracles on each side of abdominal segments V–VIII; *F*. *greeni* always has more than four tergocentral setae total on each of these segments (Guimarães, 1985; Price et al., 2008). In addition to these tergocentral setae, *F*. *pezopori*, *F*. *josephi* and *F*. *greeni* have a stronger dorsal seta associated with the spiracle on some abdominal segments, as well as a tergolateral (= paratergal) seta. In *F*. *josephi* and *F*. *greeni*, the spiracular setae are posterolateral relative to the spiracles such that Price et al. (2008) did not include these in their count of tergal setae. However, in *F*. *pezopori*, the spiracular setae are posteromedial relative to the spiracles and so would be included as tergocentral using the definition in Price et al. (2008), despite seemingly being homologous with setae otherwise treated as tergolateral. Thus, excluding the spiracular setae, *F*. *pezopori* has only two pairs of tergocentral setae on abdominal segments V–VII, like in *F*. *josephi*, although there are two or three (*vs* two) pairs on VIII. *Forficuloecus pezopori* has a spiracular seta on either side of IV–VIII and a tergolateral seta either side on VI–VII, whereas, as far as we can determine from the figures in Price et al. (2008) and Guimarães (1985), *F*. *josephi* lacks spiracular (and tergolateral) setae on IV and has both spiracular and tergolateral setae on V, and both *F*. *josephi* and *F*. *greeni* have both spiracular and tergolateral setae on VIII. *Forficuloecus pezopori* has up to nine tergocentral setae on II, with three to five either side, *vs* up to six with two to three either side in *F*. *josephi* and up to seven with two to four either side in *F*. *greeni*, plus usually an additional pair of submedial setae on II anterior to the row of tergocentral setae and not reported for *F*. *josephi* or *F*. *greeni*. *Forficuloecus pezopori* has more sternocentral setae on III–V than *F*. *josephi*, and more sternolateral setae on V–VIII than *F*. *greeni* (see Table 2). Chaetotaxy of the terminal abdominal segments appears to be similar for all three species, in both sexes, although the illustration of Price et al. (2008) suggests the female may have only about five pairs of setae on the subgential fold *vs* about nine in both *F*. *pezopori* and *F*. *greeni*.

The male terminal genitalia of *F*. *pezopori* is similar to that of both *F*. *josephi* and *F*. *greeni*. In *F*. *pezopori*, the parameres appear to be straighter and narrower relative to both species, the penis appears to be relatively straighter than in *F*. *josephi* and more similar to that of *F*. *greeni*, the endomeres are perhaps narrower than in either species and lack the minute setae depicted for *F*. *greeni*, and the shape of the posterior margin of the basal apodeme is more similar to that of *F*. *josephi*. The tergopleurites of IX in the male appear to divided in *F*. *pezopori* and *F*. *josephi vs* complete in *F*. *greeni*.

## 4 Discussion

### 4.1 Richness of Philopteridae from Australian parrots

*Forficuloecus pezopori* is the eleventh species proposed for its genus, the only louse yet known from a ground parrot (i.e., *Pezoporus* spp.), and the only metazoan parasite known from *P*. *flaviventris*. Indeed, *F*. *pezopori* is the first metazoan parasite reported from any of Australia's six endangered parrot species. Including *P*. *flaviventris*, there are 43 extant and one historically extinct species of Australian parrot (Psittaculidae), all except one of which are, or have local subspecies which are endemic (BirdLife Australia, 2022). Parasites remain unknown from many of these parrots; substantial richness clearly remains to be discovered.

Species of *Forficuloecus* are known only from Australasian parrots (Psittaculidae) and appear to have moderate to high host-specificity. Seven of the 11 species, including *F*. *pezopori*, are known only from a single host species, two are known from multiple congeneric hosts, and two are known from hosts spanning two genera (Price et al., 2008). No parrot species is yet known to host more than one species of *Forficuloecus*, however, Bourke's parrot *Neopsephotus bourkii* (Gould, 1841) is host to *F*. *josephi* as well as another philopterid *Neopsittaconirmus vincesmithi* Price & Johnson, 2007, and the bluebonnet *Northiella haematogaster* (Gould, 1838) is likewise host to two philopterids but no known species of *Forficuloecus* (Price et al., 2008; Price and Johnson, 2007). Eight of the eleven species of *Forficuloecus* are known from Australia, two only from New Zealand, and one only from New Guinea (Price et al., 2008).

### 4.2 Host-specificity of *Forficuloecus pezopori*

We presume *F*. *pezopori* is specific to *P*. *flaviventris* (western ground parrot). Until recently, *P*. *flaviventris* has been considered a subspecies of *P*. *wallicus* (eastern ground parrot). Regardless of whether the western ground parrot is recognised as a distinct species, they are substantially separated geographically from the eastern ground parrot and genetic analyses suggest the two diverged prior to the Pleistocene (Joseph et al., 2011; Murphy et al., 2011). It is therefore plausible that western and eastern ground parrots support distinct parasite fauna. A feather mite *Dubininia pezopori* Mironov, Ehrnsberger & Dabert, 2017 (Sarcoptiformes: Xolalgidae), is known from, and only from, the Tasmanian subspecies of the eastern ground parrot *P*. *wallicus leachi* Matthews, 1912 (Mironov et al., 2017). As far as we are aware, no metazoan parasites have been reported from the mainland eastern ground parrot *P*. *wallicus wallicus* (Kerr, 1972), nor from the only other ground parrot, the night parrot *P*. *occidentalis* (Gould, 1861); there is no indication that any species of *Pezoporus* has been substantially investigated for parasites.

### 4.3 Endangerment and outlook for *Forficuloecus pezopori*

Presuming *F*. *pezopori* is specific to *P*. *flaviventris*, it is therefore co-endangered and faces at least the same threat of extinction as its host. Indeed, we argue that it is likely more imperilled. Parasites are typically overdispersed among host individuals, and prevalence, distribution and longevity of an oioxenous parasite species at most match the host population/individual, but are usually lesser, and frequently substantially so. Data for *F*. *pezopori* are imperfect, but our modest sampling suggests prevalence is less than 50%. Improving estimates of prevalence and abundance for *F*. *pezopori* is not necessary, feasible or advisable: the host population is already monitored and can be used as a proxy, whereas direct estimates would require trapping and handling wild host individuals and likely lethal sampling of the lice, thereby negatively impacting both parasite and host populations. However, any handling of western ground parrots as part of ongoing field monitoring should consider incorporating a check for presence of *F. pezopori* if practical.

Persistence of the western ground parrot is likely reliant on conservation intervention (Burbidge et al., 2016, 2018), and persistence of *F*. *pezopori* is therefore also dependent on the interventions for its host. Recently, the first wild-to-wild translocation of *P*. *flaviventris* was trialled, with seven individuals (Stokes et al., 2021); it is not known whether any of those carried *F*. *pezopori*. The most important benefit of successful establishment of a second population of *P*. *flaviventris* is redundancy and this can be conferred to *F*. *pezopori* if included on translocated hosts. Philopterid lice seemingly do not typically cause substantial harm to healthy, wild host individuals, nor typically negatively impact wild host populations. Thus, we suggest that western ground parrots involved in wild-to-wild translocations ought to be checked but not treated for lice. Management of lice for individual parrots may be necessary in captive settings, such as the breeding program for *P*. *flaviventris* at Perth Zoo. However, care and management plans should consider that *F*. *pezopori* also faces imminent extinction, and infestations on captive birds therefore present an opportunity for dual host-parasite conservation.

## Funding

This research did not receive any specific grant from funding agencies in the public, commercial, or not-for-profit sectors. SBM is supported by an Australian Biological Resources Study (ABRS) National Taxonomy Research Grant (NTRG) G046WN7, an Australia and Pacific Science Foundation research grant APSF21048, and funds from the Centre for Sustainable Aquatic Ecosystems, Harry Butler Institute, Murdoch University.

## CRediT authorship contribution statement

**Storm Blas Martin:** Conceptualisation, Data curation, Formal analysis, Investigation, Methodology, Project administration, Validation, Visualisation, Writing – original draft, Writing – review & editing. **Sarah Keatley:** Data curation, Methodology, Writing – review & editing. **Alisa Wallace:** Resources, Writing – review & editing. **Rebecca J. Vaughan-Higgins:** Resources, Writing – review & editing. **Amanda Ash:** Conceptualisation, Project administration, Writing – review & editing.

## Declaration of competing interest

The authors declare no conflict of interest.
